# Cell size differences affect photosynthetic capacity in a Mesoamerican and an Andean genotype of *Phaseolus vulgaris* L.

**DOI:** 10.3389/fpls.2024.1422814

**Published:** 2024-09-11

**Authors:** Andrew Ogolla Egesa, C. Eduardo Vallejos, Kevin Begcy

**Affiliations:** ^1^ Environmental Horticulture Department, University of Florida, Gainesville, FL, United States; ^2^ Horticultural Sciences Department, University of Florida, Gainesville, FL, United States; ^3^ Plant Molecular and Cellular Biology Graduate Program, University of Florida, Gainesville, FL, United States

**Keywords:** carboxylation, common bean, gene pools, leaf anatomy, photosynthetic efficiency

## Abstract

The efficiency of CO_2_ flux in the leaf is hindered by a several structural and biochemical barriers which affect the overall net photosynthesis. However, the dearth of information about the genetic control of these features is limiting our ability for genetic manipulation. We performed a comparative analysis between three-week-old plants of a Mesoamerican and an Andean cultivar of *Phaseolus vulgaris* at variable light and CO_2_ levels. The Mesoamerican bean had higher photosynthetic rate, maximum rate of rubisco carboxylase activity and maximum rate of photosynthetic electron transport at light saturation conditions than its Andean counterpart. Leaf anatomy comparison between genotypes showed that the Mesoamerican bean had smaller cell sizes than the Andean bean. Smaller epidermal cells in the Mesoamerican bean resulted in higher stomata density and consequently higher stomatal conductance for water vapor and CO_2_ than in the Andean bean. Likewise, smaller palisade and spongy mesophyll cells in the Mesoamerican than in the Andean bean increased the cell surface area per unit of volume and consequently increased mesophyll conductance. Finally, smaller cells in the Mesoamerican also increased chlorophyll and protein content per unit of leaf area. In summary, we show that different cell sizes controls the overall net photosynthesis and could be used as a target for genetic manipulation to improve photosynthesis.

## Highlights

- Leaf photosynthetic performance comparison between a Mesoamerican (Jamapa) genotype and an Andean (Calima) genotype showed that smaller cell size and higher stomatal density found in Jamapa contributed to higher photosynthetic performance.

## Introduction

Enhancing photosynthetic efficiency can improve plant performance and productivity (Baker et al., 2007; Cardona et al., 2018; Lin et al., 2022; Keller et al., 2024). We have a limited understanding of the impact of anatomical, biochemical, and physiological architectures of the photosynthetic gas exchange apparatus on net photosynthesis (A_net_) (Baker et al., 2007; Sakoda et al., 2022). Nevertheless, recent studies have identified inter- and intra-specific phenotypic variation in photosynthetic gas exchange structures associated with adaptation to different environments (Tanaka et al., 2019; Müller and Munné-Bosch, 2021; Cackett et al., 2022; Sakoda et al., 2022). For instance, adaptation to a wide range of hydrological environments by species of *Banksia* are related to changes in morphological and anatomical characteristics that impact net assimilation (Drake et al., 2013).

Leaf traits impact plant photosynthesis by regulating the ability to use CO_2_ and light (Drake et al., 2013, 2019; Harrison et al., 2020; Elferjani et al., 2021). Manipulating stomatal characteristics can improve photosynthetic capacity (Tanaka et al., 2013; Ren et al., 2019; Harrison et al., 2020). For instance, size, shape, and density of the stomata as well as the architecture of the cellular and intercellular leaf layers, control CO_2_ diffusion through the intracellular space into the chloroplasts (Büssis et al., 2006; Drake et al., 2013; Kollist et al., 2014; McAusland et al., 2016; Peguero-Pina et al., 2017). Particularly, the mesophyll cell size and wall thickness are inversely associated with the CO_2_ diffusion path into the chloroplasts (see review by Ren et al., 2019). These traits are critical in C3 plants due to their higher susceptibility to limited CO_2_ in the chloroplasts which promotes photorespiration.

The common bean (*Phaseolus vulgaris*, L.) is the most cultivated legume used for direct human consumption (Gepts, 2001), and it represents a significant component of the protein and carbohydrate caloric intake for over half a billion people worldwide (Siddiq et al., 2011; OECD, 2019; Uebersax et al., 2022). Therefore, improving the productivity of common bean will have a significant global impact on food security. The potential for improvement is based on the extent of variation in this species. DNA sequence analysis revealed that Mesoamerica is the primary center of diversity of *P. vulgaris*, from which it radiated to the Andean region (Gaut, 2014). Furthermore, allele frequency analysis also indicated that beans were domesticated independently in each gene pool (Schmutz et al., 2014). More recently, associations of certain DNA variants of beans with ecological niches independent of geographical distributions have been reported (Rodriguez et al., 2016).

Several groups have matched the extent of genotypic diversity between the gene pools and wild and cultivated beans with the phenotypic diversity of variable traits. For instance, significant phenotypic differences in seed size and yields among common beans between Mesoamerican and Andean cultivars were reported (Sexton et al., 1997). These findings pointed out that there were also some contrasting relative growth rates between the two groups under variable environmental conditions. On the other hand, Lynch et al. (1992) and González et al. (1995) reported extensive variation in leaf morphology, anatomy, biochemistry, and assimilation rates among a relatively large set of wild accessions from both gene pools. However, there is still very limited information on the extent and impact of the diversity of the anatomical traits on domesticated common bean genotypes. Early studies established that Mesoamerican beans have smaller organ (i.e. leaves and seeds), and cell sizes than the Andean genotypes (Singh, 1981; Singh et al., 1991; Sexton et al., 1997). These peculiar distinctions have been the basis of the selection of Jamapa as a representative cultivar for the Mesoamerican gene pool and Calima as its Andean counterpart. These two genotypes have been used as parental lines for QTL analysis to understand the inheritance of variable traits of common beans (Bhakta et al., 2015, 2017; Cichy et al., 2015). Therefore, we selected these two common bean genotypes to understand photosynthetic efficiency, particularly in the era of rapid climate change. We hypothesized that some of the existing anatomical differences could explain differences in photosynthetic characteristics between the two common bean genotypes from Andean and Mesoamerican gene pools.

Genetic characterization of the existing variation in photosynthesis-associated traits between Mesoamerican and Andean beans could enable genetic manipulations of the photosynthetic apparatus. The main aim of this study was to use two distinctly variable common bean genotypes that originated from separate domestication events to investigate the extent of the influence of anatomical and morphological traits on their photosynthetic gas exchange sites and how they impact photosynthesis in variable conditions of light and CO_2_. Therefore, we used Calima, domesticated in the Andean region, and Jamapa, domesticated in the Mesoamerican region, and we examined their differential patterns of carbon assimilation responses to light and CO_2_ and how these variable patterns could be explained by their anatomical differences.

## Materials and methods

### Plant materials and growth conditions

We selected for comparative analysis a representative genotype from each of the two *Phaseolus vulgaris* L. gene pools. Jamapa is a small, black-seeded landrace from Mesoamerica with an indeterminate growth habit, and Calima is a mottled large-seeded Andean bean with a determinate growth pattern (Egesa et al., 2024b). In addition, these genotypes exhibit contrasting photoperiod sensitivity (Bhakta et al., 2017), and are the parents of a recombinant inbred family (Bhakta et al., 2015).

Seeds from both genotypes were germinated in a 72-well nursery tray custom black (Nursery Supplies, FL) and transplanted at ten days into one-gallon black molded nursery cans. Three-week-old plants from each genotype were used for all the experiments. This age was selected to avoid other potential confounding effects such as differences in the timing of juvenility-to-maturity transitions as well as the rapid sink-source tissue relocation of photo-assimilates normally happening during reproduction. The media used in the nursery and transplanting was PRO-MIX HP Mycorrhizae planting media (Premier Horticulture, Canada). After transplanting, 17 g of Osmocote (N:P: K 18:6:12) were added to each pot. Greenhouse temperatures were maintained at 26±3 °C/20±3 °C day/night respectively, relative humidity of 50±5% and daylight illumination of 1000±200 μmol m^-2^ s^-1^ photosynthetic photon flux density (PPFD). Irrigation was provided daily by applying water to the field capacity.

### Physiological measurements

The uppermost completely expanded mature trifoliate leaf of each genotype was used to measure CO_2_ assimilation (A), transpiration rate (E), stomatal conductance to water vapor (g_sw_), intercellular CO_2_ (C_i_), and total conductance to CO_2_ (g_tc_), using the LI-COR Li-6800 machine (Begcy et al., 2019; LI-COR, 2023). The temperature inside the leaf chamber was maintained at 25°C.

### CO_2_ conductance measurements

We exposed the mature trifoliate leaf of each genotype to three levels (200, 400, and 600 µmol mol^-^¹) of CO_2_ in the Li-6800 leaf chamber using a photosynthetic photon flux density (PPFD) of 1000 μmol m^-2^ s^-1^ and 25°C as set temperature. We then compared the patterns of leaf conductance using the measured values of A, E, g_sw_, C_i_, and g_tc_. We then used A, C_a_, and C_i_ values to estimate the CO_2_ conductance from outside into the leaves using the formula g_c_=A/(C_a_-C_i_) (Boyer and Kawamitsu, 2011).

### Light response curves

Responses to light were measured at 25°C and relative humidity of 60% ± 2% at two levels of CO_2_: ambient (400 µmol mol^-^¹) and elevated (600 µmol mol^-^¹) CO_2_. The light levels were gradually increased from 0 to 1800 μmol m^-2^ s^-1^ (PPFD) (Begcy et al., 2019). The second level of CO_2_ (600 µmol mol^-^¹) in the experiment was to test the potential impact of the rising CO_2_ levels on the two common bean genotypes. The measurements were collected in the morning from 8 to 10 am, during the midday from 11 am to 1 pm and in the afternoon, from 2 to 4 pm.

### CO_2_ response (A−Ci) curves

CO_2_ response (A−Ci) curves were obtained at moderate PPFD (1000 μmol m^−2^s^−1^) for both bean genotypes. The ambient CO_2_ (C_a_) was adjusted between 50 and 600 µmol mol^-^¹. The measurements were collected in the morning from 8 to 10 am, during the midday from 11 to 1 pm and in the afternoon, from 2 to 4 pm (afternoon) V_cmax_ and J_max_ were estimated using a modified Farquhar‐von Caemmerer‐Berry model as described in the plantecophys package (Duursma, 2015).


$$Am=\frac{Ac+Aj-\sqrt{{\left(Ac+Aj\right)}^{2}-4\theta AcAj}}{2\theta }-R_{d}$$

Where:

A_m_ = the hyperbolic minimum of A_c_ and A_j_.

A_n_ = min (A_c_, A_j_) - R_d_.

A_n_ = Net CO_2_ assimilation.

A_c_ = Photosynthesis rate when Rubisco activity is limiting.

A_j_ = Photosynthesis rate when RuBP –regeneration is limiting.

R_d_ = the rate of mitochondrial respiration. θ =theta = 0.85.

A_c_, the rubisco-limited photosynthesis rate was estimated as previously described (Duursma, 2015), and estimated as:


$$A_{c}=V_{cmax}\left(C_{i}-\Gamma^{*}\right)/\left[C_{i}+K_{c}\left(1+O_{i}/K_{o}\right)\right]$$

Where V_cmax_ is the maximum rate of Rubisco activity, C_i_ and O_i_ are the intercellular concentrations of CO_2_ and O_2_, K_c_ and K_o_ are the Michaelis–Menten coefficients of Rubisco activity for CO_2_ and O_2_, respectively, and Γ* is the CO_2_ compensation point in the absence of mitochondrial respiration.

A_j_, the photosynthesis rate when ribulose-1,5-bisphosphate (RuBP)-regeneration is limiting was estimated as previously described (Laĭsk et al., 2009; Duursma, 2015), and according to:


$$A_{j}=\left(J/4\right)\times \left(Ci-\Gamma^{*}\right)/Ci+2\;\Gamma^{*})$$

Where J is the rate of electron transport which is related to incident photosynthetically active photon flux density, Q, by:


$$qJ^{2}-\left(aQ+J_{max}\right)J+aQJ_{max}=0\left(when\;J<J_{max}\right)$$

where;

q = is the quantum energy state.

a = absorbance by leaf photosynthetic pigments.

We modeled the A-Ci curves using the Duursma approach to estimate the photosynthesis rate for ribulose-1,5-bisphosphate (RuBP) saturated and RuBP-regeneration limited conditions (Duursma, 2015).

### Chlorophyll quantification

A set of leaf discs measuring 2.01 cm^2^ from fresh leaf tissue were harvested from each genotype. After fresh weight determination, discs were finely ground in liquid nitrogen and dissolved in four volumes of 100% of ice-cold acetone. The homogenate was brought up to 1 mL with 80% ice-cold acetone and mixed by vortexing for 20 seconds. The mixture was centrifuged at 20,000 g for 5 minutes, and the supernatant was obtained. Afterward, 150 μL of the chlorophyll extract was used to read the absorbance using a plate reader at 645nm and 663nm wavelengths to estimate chlorophyll a (ChlA) and chlorophyll b (ChlB), respectively. Total chlorophyll was calculated by the sum of Chlorophyll a and Chlorophyll b as described previously (Warren, 2008; Begcy et al., 2012).

### Total protein quantification

An additional set of leaf discs measuring 2.01 cm^2^ each from fresh leaf tissue were harvested for the total protein quantification. Discs were finely ground in liquid nitrogen and dissolved with equal volume (v/v) of the 2X Protein Extraction buffer (PE buffer: 0.1 M tris-HCl, pH 8; 2% SDS; 0.05 mL 1M DTT). Followed by the addition of 1X PE buffer to obtain a 1 mL sample-PE buffer mixture before further mixing by vortexing and progressing with the protein extraction. The mixture was then heated in a water bath at 100°C for 10 minutes, then allowed to cool at room temperature for 10 minutes, then pelleted at 20,000 g for 10 minutes at 23±1°C. 200 μL of the supernatant was transferred to new centrifuge tubes and mixed with 800 μL of 100% acetone. The mixture was centrifuged at 20,000 g at 23±1°C for 10 minutes and the supernatant was discarded. The pellet was allowed to dry at room temperature for 2 mins, then dissolved in 50 μL of 0.2 N NaOH and neutralized with an equal volume of 0.2 N HCl. The total protein content was determined using the colorimetric Bio-Rad Protein Assay Kit II (Bio-Rad Laboratories, CA). Seven dilutions of a protein standard containing 0 to 30 µg/mL of the total protein content were used. A standard curve was prepared each time the assay was performed. The absorbance at 595 nm (A595nm) was then measured with a microplate spectrophotometer (Epoch Microplate Spectrophotometer; BioTek, Winooski, VT), and the normalized absorbance values were plotted versus the mass concentration (µg of protein/mg of leaf tissue) as previously described (Kalaman et al., 2022; Egesa et al., 2024a).

### Stomatal density

To quantify stomatal density, plants were transferred from the greenhouse to the lab (light ~ 10 μmol m^−2^s^−1^ PPFD). Then, leaf samples were prepared using the modified leaf peel method (Lawrence et al., 2018). In brief, intact leaves were carefully covered with clear adhesive tape on the abaxial and adaxial sides to obtain a leaf tissue peel with intact cuticle, epidermal, and guard cells. Before imaging, the peels were kept moist in 1% PBS and a small area (5 cm by 1.5 cm) was excised and mounted on a glass slide. A total of 34 plants per genotype were used and 10 excised peels were taken per plant from the abaxial and the adaxial side. Imaging was performed on a Leica compound microscope (Wetzlar, Germany) at a magnification of 10X. The microscope was fitted with Leica microsystems CMS camera calibrated with Leica Application Suite X LAS X (3.7.4.23463) for imaging. We used 25 µL of 5% propidium iodide to enhance the boundaries of the epidermal and guard cells. The images depicted the pavement cells with the closed stomata. The image J software (Schneider et al., 2012) was then used to determine stomatal density and leaf epidermal cell sizes.

### Stomata and guard cell size estimation

To quantify stomata size, guard cells size, and fully open stomata aperture area, detached fresh leaf samples were incubated in 150 mL of stomata opening buffer (50 mM KCl, 10 mM MES-KOH, pH 6.2) (Lawrence et al., 2018) for 30 min. Following incubation, a section (5 cm by 1.5 cm) was excised and mounted on a glass slide. Imaging was performed on a Leica compound microscope (Wetzlar, Germany) at magnifications of 40X objective lens. We used 34 plants from each genotype and obtained 10 images from the Abaxial and the adaxial side of the leaf per plant. The images obtained from each genotype were used to determine the size of stomata, guard cells, and full stomata aperture area using the image J software (Schneider et al., 2012).

### Cell layer measurements

Fresh leaf samples from three-week-old plants from each genotype were used to prepare cross-sections by hand. Sections were mounted on glass slides using thin forceps. The sections were stained with 25 μL of 5% propidium iodide and imaged at 10X objective lens on a Leica compound microscope (Wetzlar, Germany). Images were used to count the number of cell layers using the Leica microsystems CMS camera calibrated with Leica Application Suite X LAS X (3.7.4.23463).

### Palisade and mesophyll cell size characteristics

Fresh leaf samples from three-week-old plants were obtained from each genotype and used for cell isolation. The palisade and mesophyll cells were isolated using a modified leaf cell isolation protocol (Endo et al., 2016). Excised leaf discs (0.5-inch diameter) with their epidermal cell layer peeled off were incubated in 1.7 mL Eppendorf tubes containing 1 mL of ice-cold cell isolation enzyme buffer (75% (wt/vol) cellulase ‘Onozuka’ R-10, 0.25% (wt/vol) macerozyme R-10, 0.4 M, mannitol, 8 mM CaCl_2_ and 5 mM MES-KOH) for 20 minutes on a rotating Biometra OV4 Compact Line Hybridization Oven Incubator set at 24°C. Macerated discs were removed from the Eppendorf tubes before centrifugation at 200 g for 5 min at 4°C. The supernatant was discarded, then the isolated cells were gently re-suspended in 500 μL of the ice-cold wash buffer (2 mM MES,125 mM CaCl_2_,154 mM NaCl, 5 mM KCl) before another round of centrifugation at 200 g for 5 min at 4°C. The supernatant was discarded, and the pellets were resuspended in 100 μL of the cell flotation buffer (4 mM MES, 0.4 M mannitol, and 15 mM MgCl_2_ at pH 5.7) (Yoo et al., 2007; Nanjareddy et al., 2016), before imaging on a compound microscope at 40X objective lens.

Dimensions of the isolated cells were obtained using the Image J software (Schneider et al., 2012). Side projections of palisade cells were used to obtain the diameter (D), radius (r=D/2), and length (L). The volume of palisade cells was estimated as v = πr^2^L, and the surface area was estimated as SA = 2πr^2^ + 2πrL. Spongy mesophyll cell size was calculated by estimating an average radius of a sphere from three diameter estimates (d1, d2, d3) then the volume was estimated as V= 4/3πr^3^ and the surface area was estimated as SA = 4πr^2^.

### Statistical analysis

Differences between photosynthetic parameters, anatomical and physiological traits of the two genotypes were statistically tested using the Welch’s t-test (α = 0.05). The CO_2_ response data was subjected to Farquhar—von Caemmerer—Berry using the FvCB model for C_3_ photosynthesis as implemented by Duursma (2015). The model was used to estimate V_cmax_, J_max_, R_d,_ and to determine the intercellular CO_2_ levels at which the carboxylation-limited to RuBP regeneration-limited photosynthesis occurred.

## Results

### Genotypes from the Andean and Mesoamerican gene pools display different photosynthetic performance under high light and CO_2_ conditions

**Figure 1:**
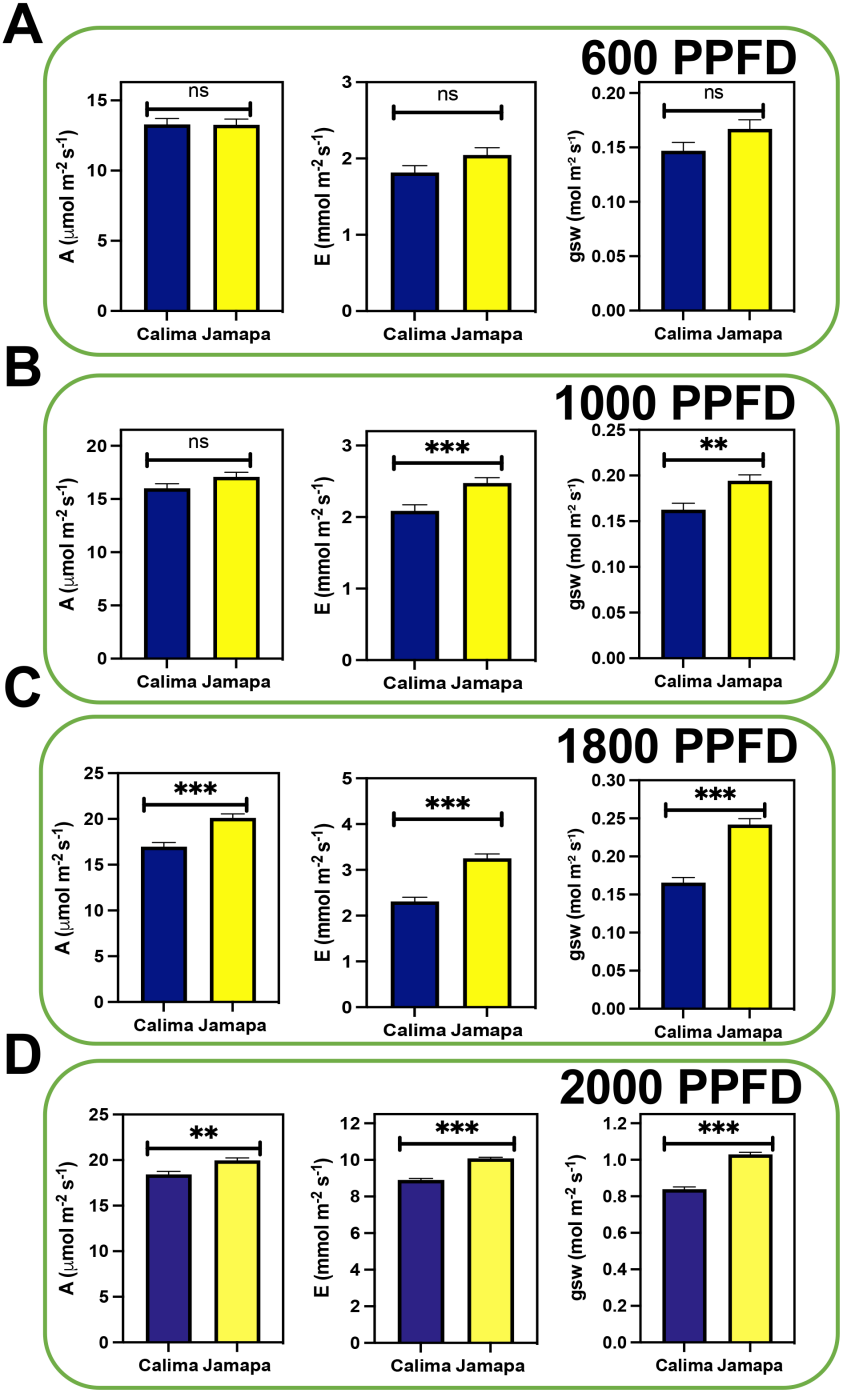
Photosynthetic capacity increases in the Mesoamerican genotype under higher light intensity. Photosynthetic gas exchange parameters (Assimilation [A], Transpiration [E], and Stomata conductance to water vapor [gsw]) were measured under different levels of light intensity at ambient CO_2_ (400 µmol mol^-1^). The light levels comprised Photosynthetic Photon Flux Density (PPFD) levels of **(A)** 600 µmol m^-2^ s^-1^, **(B)** 1000 µmol m^-2^ s^-1^, **(C)** 1800 µmol m^-2^ s^-1^ and **(D)** 2000 µmol m^-2^ s^-1^. n = 20. Significant differences were calculated based on Welch’s t-test at an alpha of 0.05. Non-significant (ns) P > 0.05; **P ≤ 0.01, and *** P ≤ 0.001.

To characterize the influence of transient changes in light intensity on photosynthetic capacity in *P. vulgaris*, we used two common bean genotypes from the Andean (Calima) and Mesoamerican (Jamapa) gene pools. First, we subjected both genotypes to low and moderate light intensity, 600 and 1000 µmol m^-2^ s^-1^ Photosynthetic Photon Flux Density (PPFD), respectively (**Figures 1A, B**). We did not find significant differences between Jamapa and Calima in their photosynthetic levels (A). However, the transpiration rate (E) and stomatal conductance (gsw) levels were significantly higher in Jamapa at moderate light levels (**Figure 1B**). At a higher light intensity, specifically 1800 µmol m^-2^ s^-1^ (PPFD) (**Figure 1C**) and 2000 µmol m^-2^ s^-1^ (PPFD) (**Figure 1D**), Jamapa showed consistently statistically significant higher A, E and gsw than Calima. Based on their origin, these results suggest that both genotypes have adapted to different light intensities, with Calima adapting to the low light while Jamapa to high light intensity.

### Diurnal patterns of net assimilation in the Andean and Mesoamerican genotypes

**Figure 2:**
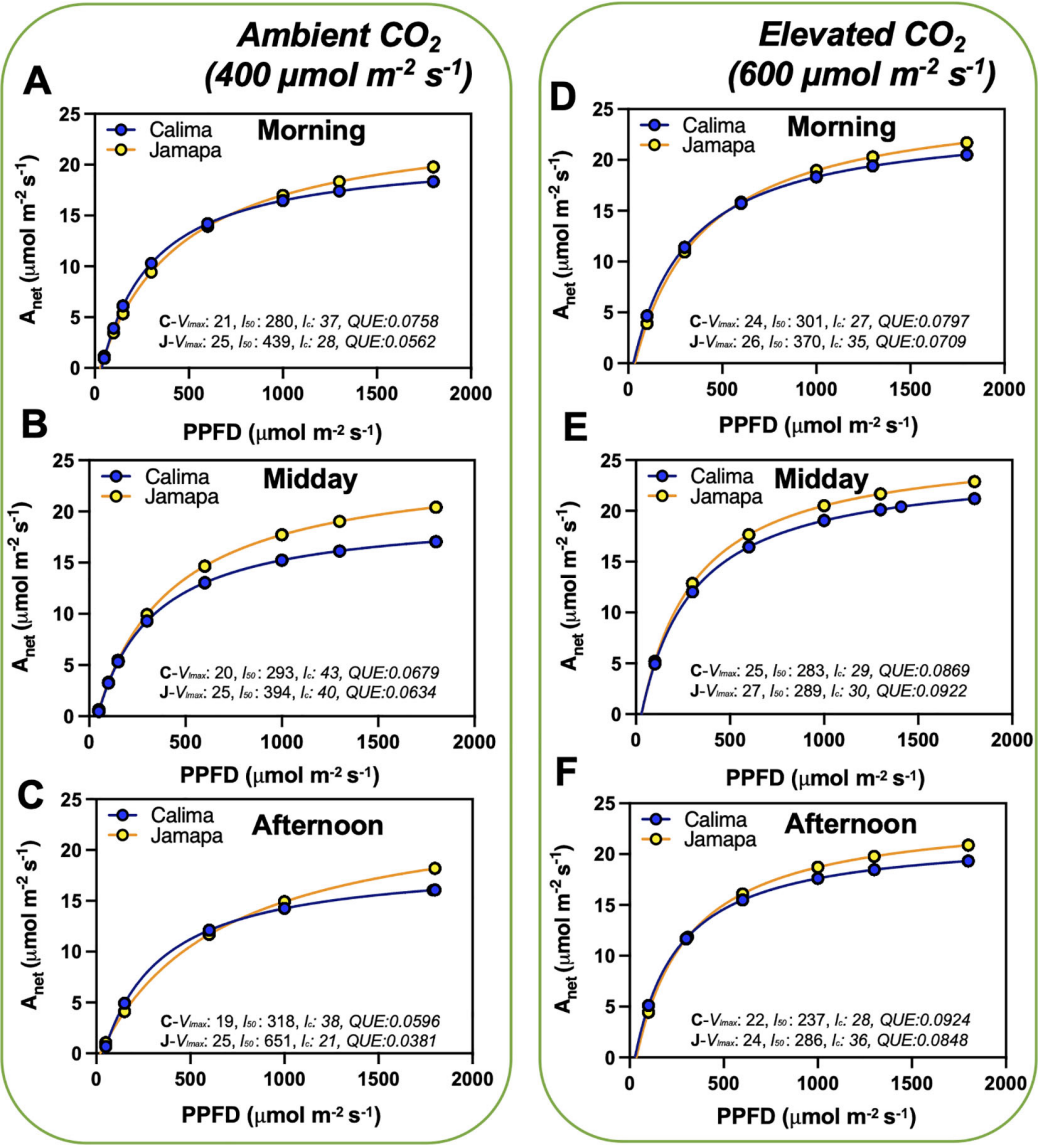
Diurnal photosynthetic light use efficiency characteristics are variable from Andean and Mesoamerican common bean genotypes. We used a modified hyperbolic function from light response curves (LRCs) to estimate the light compensation points (*l_c_*; x-axis intercept), the maximum photosynthetic rate at light-saturating conditions (*V_lmax_*; horizontal asymptote), the *l_50_* or PPFDs needed to attain 0.5 *V_lmax_* and the quantum use efficiency (QUE= ΔA/ΔPPFD). These data were collected from 8 to 10 am (morning), 11 to 1 pm (midday), and 2 to 4 pm (afternoon) **(A-C)** ambient CO_2_ (400 µmol mol^-1^) and at **(D-F)** elevated CO_2_ (600 µmol mol^-1^). n = 36.

To further characterize the impact on transient light adaptation of these two common bean genotypes, we analyzed their light compensation point and maximum rate of light-unlimited photosynthesis by using light curve measurements at ambient (400 µmol mol^-1^) and elevated (600 µmol mol^-1^) CO_2_ levels. We subjected both genotypes to increasing light intensity levels from 0 to 1800 µmol m^-2^ s^-1^ PPFD (**Figure 2**). We used a modified hyperbolic function for the light response curve (LRCs) using data from Calima and Jamapa to estimate the light compensation points (l_c_; x-axis intercept), the maximum photosynthetic rate at light-saturating conditions (V_lmax_; horizontal asymptote), the l_c_ or PPFDs needed to attain 0.5 V_lmax_ and the quantum use efficiency (QUE= ΔA/ΔPPFD). In general, Jamapa exhibited a higher V_lmax_ compared to Calima at both ambient and elevated CO_2_ (**Figure 2**). The light compensation points (l_c_) for both genotypes were consistently lower in Calima (**Figures 2A-C**). However, a shift from ambient to elevated CO_2_ resulted in a considerable drop in the light compensation point for both Calima and Jamapa (**Figures 2D-F**).

### Higher carboxylation and electron transfer efficiencies in the Mesoamerican genotype

**Figure 3:**
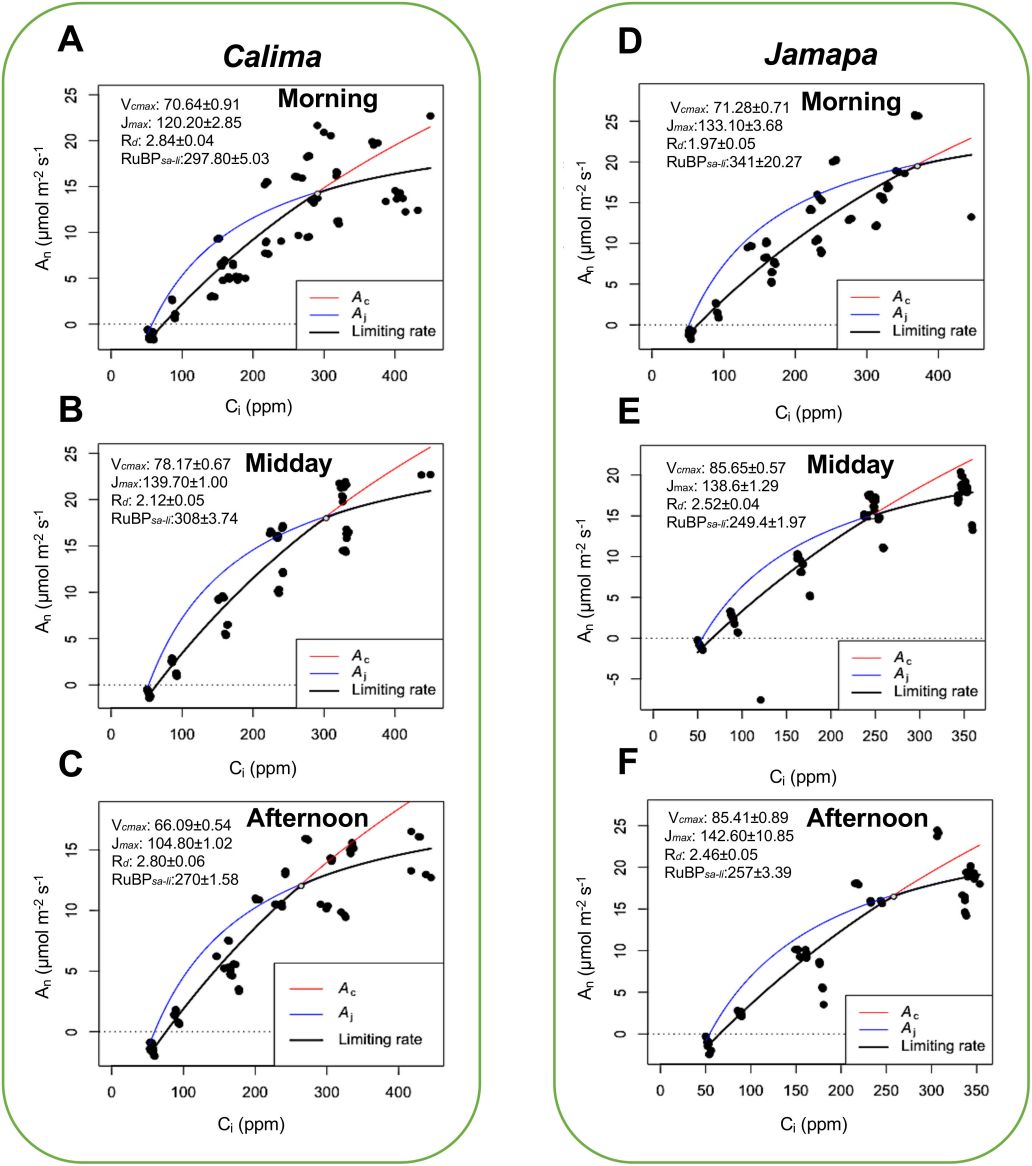
Diurnal patterns of carboxylation and electron transfer efficiencies are consistently higher in the Mesoamerican genotype and peak at different periods of the day. Maximum carboxylation (V_cmax_), Maximum rate of photosynthetic electron transport (J_max_), rate of dark respiration (R_d_), the amount of CO_2_ for the transition from ribulose-1,5-bisphosphate saturated to limited (RuBP_sa-li_) photosynthesis was estimated by fitting the A-Ci curves from photosynthesis data collected from Calima and Jamapa, where CO_2_ is the substrate in the reaction adopting the Farquhar—von Caemmerer—Berry (The FvCB model for C3 photosynthesis) as described in Plantecophys package (Duursma, 2015). Estimated carboxylation electron transfer efficiencies, dark respiration, and CO_2_ levels for RuBP_sa-li_ transition of Calima in the morning **(A)**, midday **(B)**, and afternoon **(C)**, and Jamapa in the morning **(D)**, midday **(E)**, and afternoon **(F)**. Photosynthesis data was collected at 1000 µmol m^-2^ s^-1^ PPFD, temperature of 25 °C, and relative humidity of 60%. n = 36.

To further characterize CO_2_ conductance and carboxylation efficiencies of Calima and Jamapa, we subjected both genotypes to gradually changing levels of CO_2_ (A-Ci curve), using CO_2_ levels ranging from 50 to 600 μmol mol^-1^. We estimated the maximum carboxylation (V_cmax_), Maximum rate of photosynthetic electron transport (J_max_), rate of dark respiration (R_d_), the amount of CO_2_ for the transition from ribulose-1,5-bisphosphate saturated to limited (RuBP_sa-li_) photosynthesis (**Figure 3**). Jamapa exhibited a higher Carboxylation rate (V_cmax_) and a higher linear electron transfer rate (J_max_) compared to Calima. These results were likely due to the prevailing e-transfer rate or better CO_2_ conductance across the stomata and through the mesophyll cells in Jamapa compared to Calima. Since efficient CO_2_ diffusion into the chloroplast envelopes can significantly reduce the potential of photorespiration, promoting higher net photosynthesis in a plant.

Calima’s V_cmax_ increased from the morning to midday and then dropped in the afternoon, and the J_max_ values changed in a similar fashion. Dark respiration in Calima fluctuated between 2.12 and 2.84 µmol m^-2^ s^-1^ throughout the day with an apparent dip at mid-day (**Figures 3A-C**). The intercellular CO_2_ concentration at which Calima switched from carboxylation-limited to RuBP-limited photosynthesis was stable in the morning and midday but decreased in the afternoon by 38 μmol mol^-1^ (**Figures 3A-C**). In contrast, Jamapa’s V_cmax_ increased from morning to midday from 71.28 to 85.65 μmol m^-2^ s^-1^, and remained stable in the afternoon, while its J_max_ had a net increase throughout the day (**Figures 3D-F**). Jamapa’s dark respiration fluctuated between 1.97 in the morning to 2.52 μmol m^-2^ s^-1^ in the afternoon (**Figures 3D, E**). Interestingly, Jamapa transitioned from RuBP-saturated to RuBP-limited CO_2_ assimilation at a C_i_ of 341 μmol mol^-1^ in the morning and a C_i_ of 249 μmol mol^-1^ at midday, then remained stable through the afternoon (**Figures 3D-F**). In general, the V_cmax_ and J_max_ values of Jamapa were larger than those of Calima throughout the day, except for J_max_ at midday (**Figures 3A-F**). However, the greatest difference between these genotypes was the daily dynamics of the transitions from RuBP saturated to RuBP limited photosynthesis (**Figures 3B, E**). The ratio of J_max_/V_cmax_ in Calima changed very little over the day, which was reflected in the narrow range of the C_i_’s at which the transition occurred. In Jamapa this ratio dropped from 1.9 to 1.6 throughout the day. Increased photosynthesis rate with increasing light in Jamapa compared to Calima suggested a better capture of light energy by the leaf which may have increased the availability of e^-^ and H^+^ to drive the photosynthesis reactions. These results indicated that Jamapa had a greater capacity to regenerate RuBP in the morning and that this capacity decayed during the day, in contrast to Calima, which, comparatively, did not display such a dramatic change.

### Leaf anatomy as a predictor of photosynthetic efficiency in Calima and Jamapa

We hypothesized that some of the differences in photosynthetic characteristics observed between the two common bean genotypes could be explained by their anatomical differences. To test this hypothesis, we performed comparative anatomical analyses of the leaf epidermis and the mesophyll.

**Figure 4:**
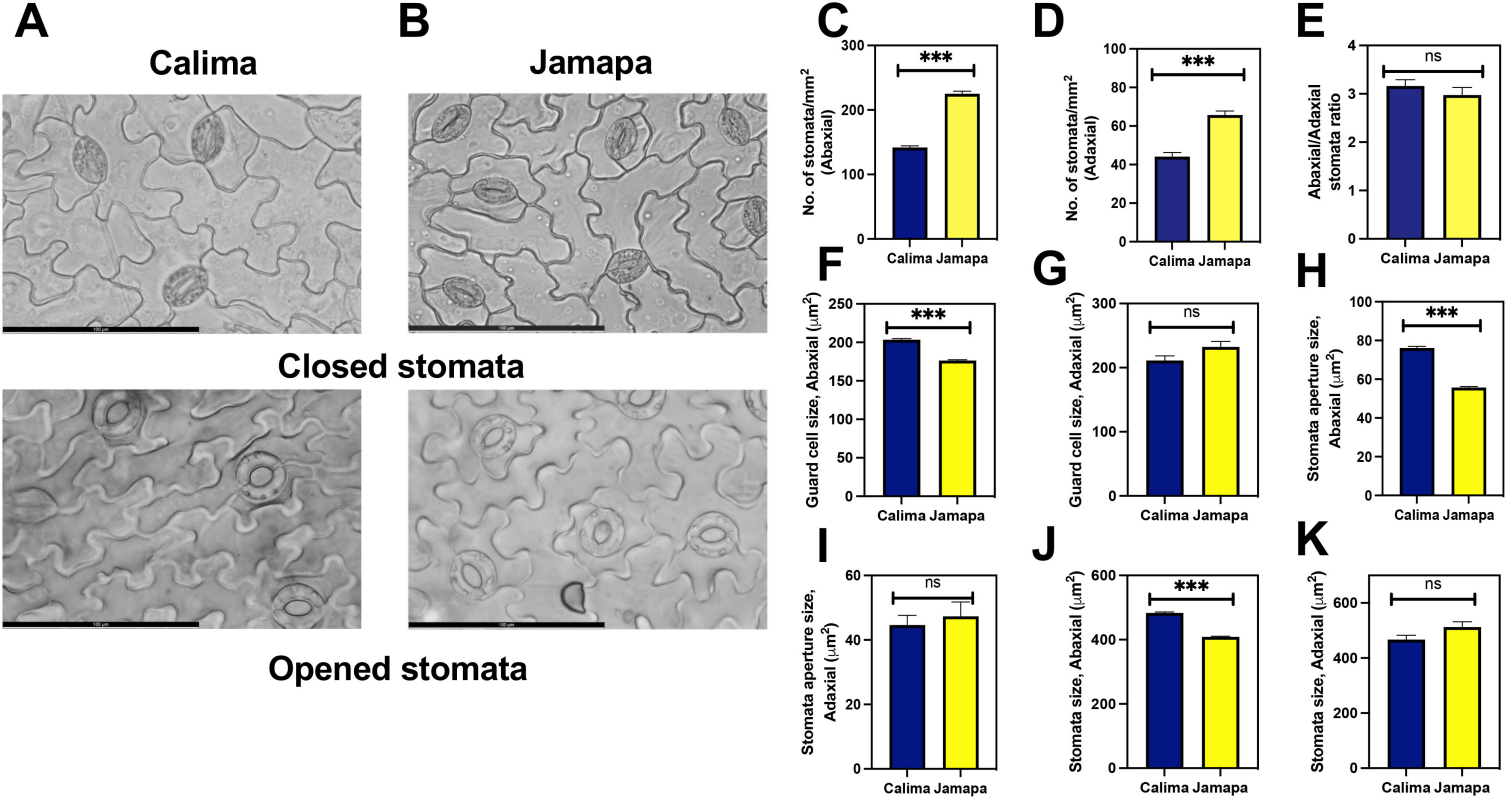
Andean common bean genotype exhibits lower stomatal density per unit area than its Mesoamerican counterpart. Light microscopy images of **(A)** Calima and **(B)** Jamapa depicting closed and opened stomata. Number of stomata on the **(C)** abaxial and **(D)** abaxial sides of the leaf. **(E)** Ratio of stomata per unit area on the abaxial to the adaxial side. Guard cell size on the **(F)** abaxial and the **(G)** adaxial side of the leaf. Open stomata aperture size on the **(H)** abaxial and the **(I)** adaxial side. Stomata size on the **(J)** abaxial and the **(K)** adaxial sides. Fresh mature leaves from three-week-old plants were incubated in 150 mL of stomata opening buffer for 30 mins to open the stomata. Significant differences were calculated based on Welch’s t-test at an alpha of 0.05. ns P > 0.05 and *** P ≤ 0.001. n = Abaxial: 346, Adaxial: 340. Scale bar = 100 μm.

First, we calculated the stomatal density using closed and opened stomata in Calima (**Figure 4A**) and Jamapa (**Figure 4B**). The stomatal density on the abaxial side of Jamapa leaves was 225±4.12/mm^2^ (**Figure 4C**) and 66±2.08/mm^2^ (**Figure 4D**) on the adaxial side. In contrast, the corresponding densities for Calima were 141±2.44/mm^2^ and 44±2.14/mm^2^ (**Figures 4C, D**). Both genotypes exhibited comparable abaxial to adaxial density ratios – 3.4 and 3.2 for Calima and Jamapa, respectively – and consequently similar intergenotypic ratios (**Figure 4E**). As a proxy to guard cell sizes, we measured the projected surface areas. On the abaxial side, Calima’s guard cells (204±1.35 μm^2^) were 15% larger than those of Jamapa (176±0.98 μm^2^) (**Figure 4F**). On the adaxial side of the leaf, the estimated surface area of Calima’s guard cells (211.0±7.42 μm^2^) was not significantly different from those of Jamapa (232±8.56 μm^2^) (**Figure 4G**). Furthermore, the apertures of fully open Calima stomata (76±0.81 μm^2^) on the abaxial side were 37% larger than those of Jamapa (55±0.51μm^2^) (**Figure 4H**). However, on the adaxial side the stomata apertures were not significantly different, Calima 44.6±2.99 μm^2^ and Jamapa 47.32±4.48 μm^2^, data that agree with the guard cell sizes (**Figure 4I**). Moreover, the stomata size was significantly larger in Calima than Jamapa on the abaxial side (**Figure 4J**), but not in the adaxial side (**Figure 4K**).

**Figure 5:**
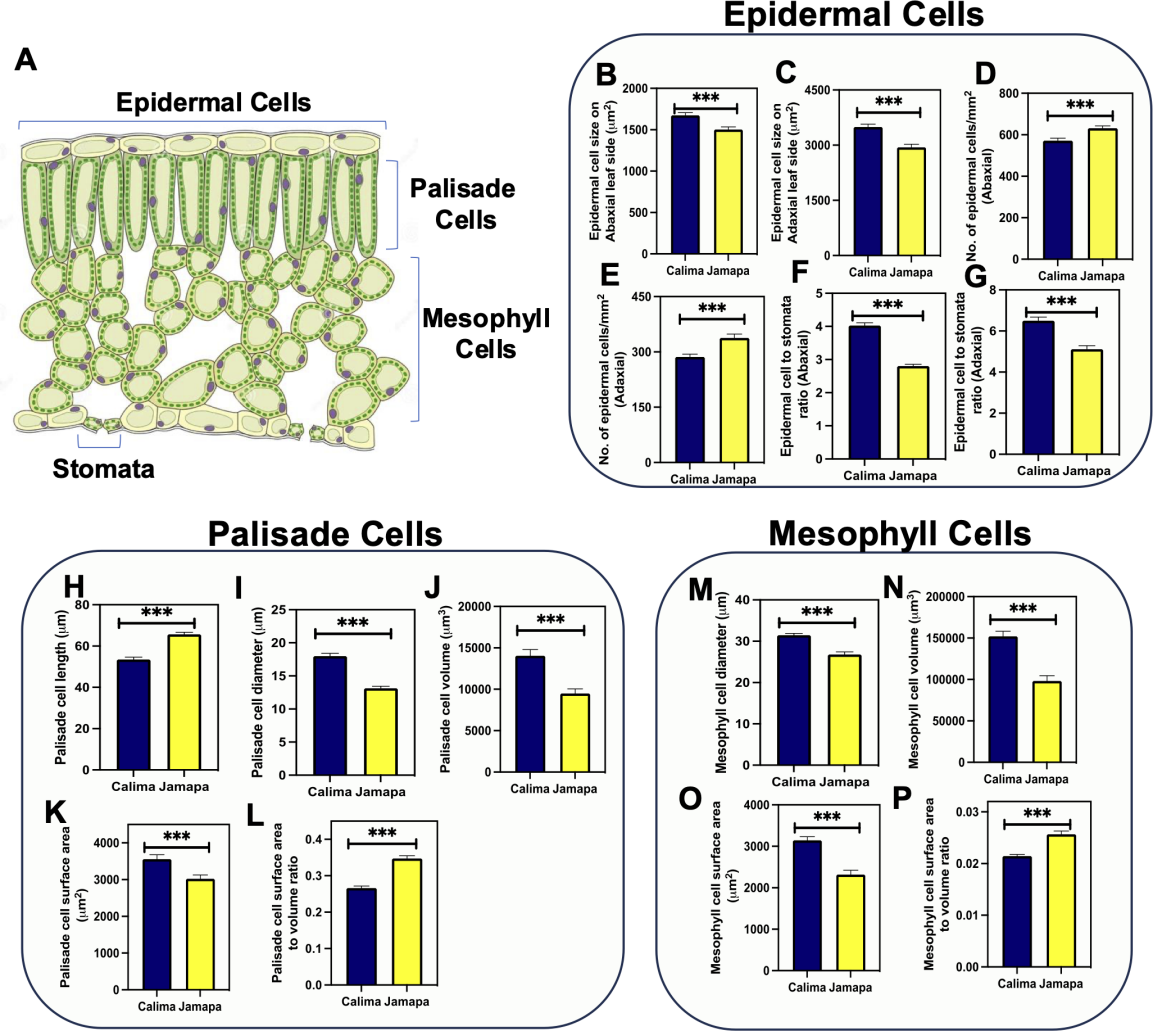
Epidermal, palisade, and mesophyll cell size is different between the Andean and the Mesoamerican genotypes. **(A)** Schematic representation of a cross section of a leaf indicating the location of stomata, epidermal, palisade and mesophyll cells. Epidermal cell sizes on the **(B)** abaxial and **(C)** adaxial sides. Number of epidermal cells on the **(D)** abaxial and **(E)** adaxial sides. Epidermal cells to stomata ratio on the **(F)** abaxial and **(G)** adaxial side in Calima and Jamapa. Palisade cells **(H)** length, **(I)** diameter and **(J)** volume. Palisade cells surface area **(K)** and surface area to volume ratio **(L)**. Mesophyll cells **(M)** diameter, **(N)** volume, **(O)** total surface area and **(P)** surface area to volume ratio. Lateral projections of palisade cells were used to obtain the diameter **(D)**, radius (r=D/2), and length **(L)**. The volume of palisade cells was estimated as v = πr^2^L, and the surface area was estimated as SA = 2πr^2 +^ 2πrL. Spongy mesophyll cell size was calculated by estimating the average radius of a sphere. The surface area was estimated as SA = 4πr^2^. Significant differences were calculated based on the Welch’s t-test at an alpha of 0.05. *** P ≤ 0.001. n = 45.

To further elucidate whether the differences in photosynthetic capacity could be explained by their anatomical differences, we measured epidermal, palisade and mesophyll cells from both genotypes (**Figure 5A**). First, we analyzed the size of the epidermal pavement cells (**Figure 5B**). The average surface area of abaxial and adaxial pavement cells of Jamapa leaves was 1,502±31 and 2,940±82 μm^2^ and those of Calima 1,674±32 and 3,493±78 μm^2^, respectively (**Figures 5B, C**). Thus, Calima pavement cells were 11 to 18% larger than Jamapa cells. Furthermore, we quantified the number of epidermal cells on the abaxial (**Figure 5D**) and the adaxial side (**Figure 5E**). Jamapa had higher quantity in both. An anatomical normalization or calculation of the number of pavement cells per stoma showed while Jamapa had 3 pavement cells per stoma on the abaxial side and 5 pavement cells per stoma on the adaxial side (**Figures 5F, G**), the corresponding ratios in Calima were 4 and 6 (**Figures 5F, G**). In summary, physically and anatomically, Jamapa had a higher stomatal density than Calima.

Examination of a cross-section of the leaf blade showed that the two genotypes had three layers of spongy parenchyma cells arranged below a single palisade cell layer. We then isolated leaf palisade and spongy parenchyma cells, measured their lateral projections and used them to obtain first-order approximations of their cell volume and surface area (**Supplementary Figure S1**). Calima palisade cells (53.52±1.10 µm) were shorter than Jamapa cells (65±0.96 µm) (**Figure 5H**), but significantly wider (17±0.42 µm) than Jamapa cells (13.14±0.3 µm) (**Figure 5I**). These dimensions were used to estimate cell volumes (**Figure 5J**), and surface areas (**Figure 5K**) assuming palisade cells as cylindrical and spongy parenchyma cells as spherical bodies. The average volume of Calima palisade cells (14,038±760.6 µm^3^) was 32% larger than those of Jamapa (9,482±544.1µm^3^) (**Figure 5J**), and the average surface area for Calima cells (3,556±122.4 µm^2^) was 14.99% larger than that of Jamapa cells (3,023±102.8 µm^2^) (**Figure 5K**). Using these values, we calculated the surface-to-volume ratio, and the results showed that Jamapa cells had 23% more surface area than Calima cells per unit of volume in the palisade cells (**Figure 5L**).

On the other hand, the average diameter of spongy parenchyma cells was larger in Calima (31.45±0.43 µm) than in Jamapa (26.79±0.63 µm) (**Figure 5M**). Similarly, the average volume of spongy parenchyma cells of Calima (152,037±6,624 µm^3^) was 35% larger than those of Jamapa cells (98,185±6,421 µm^3^) (**Figure 5N**), and the surface area of Calima cells (3,146±85.50 µm^2^) was 26% greater than that of Jamapa cells (2,318±104.8 µm^2^) (**Figure 5O**). Similarly, Jamapa cells had 19% more surface area per unit of volume in their spongy parenchyma than Calima cells (**Figure 5P**). Collectively, these results indicated that mesophyll cells in Jamapa had a larger surface area for CO_2_ diffusion than those of Calima.

### Effects of anatomical differences on physiological parameters

Following the A-Ci results, we considered the gas exchange parameters at three atmospheric CO_2_ concentrations (low C_a_ = 200, ambient C_a_ = 400, and high C_a_ = 600 μmol mol^-1^) to assess the effect of the anatomical differences on gas exchange characteristics (**Table 1**). Both genotypes displayed similar responses to the different C_a_ levels. Stomatal conductance to water vapor (g_sw_) increased when C_a_ was raised from low to ambient CO_2_, but decreased when C_a_ reached the highest level. Changes in E mirrored changes in stomatal conductance to water vapor (g_sw_). However, Jamapa displayed statistically significant greater g_sw_ and higher E rates than Calima at all C_a_ levels.

**Table 1:** Leaf assimilation and conductance in Calima and Jamapa.

Parameter	Genotype	200 μmol mol^-1^ CO_2_	400 μmol mol^-1^ CO_2_	600 μmol mol^-1^ CO_2_
A (μmol m^-2^ s^-1^)	Calima	6.20±0.23^b^	15.42±0.48^b^	17.81±0.45^a^
	Jamapa	8.04±0.27^a^	17.16±0.32^a^	18.22±0.37^a^
C_i_ (μmol mol^-1^)	Calima	161.10±1.14^b^	306.48±3.67^b^	458.72±5.28^b^
	Jamapa	165.01±1.27	327.44±3.75	494.80±6.47 ^a^
g_sw_ (μmol m^-2^ s^-1^)	Calima	0.34±0.01^b^	0.38±0.02^b^	0.28±0.02^b^
	Jamapa	0.51±0.02^a^	0.65±0.03^a^	0.50±0.03^a^
E (mmol m^-2^ s^-1^)	Calima	4.14±0.15^b^	4.45±0.2^b^	3.47±0.18^b^
	Jamapa	5.64±0.17^a^	6.51±0.25^a^	5.33±0.28^a^
g_tc_ (mol m^-2^ s^-1^)	Calima	0.20±0.0077^b^	0.22±0.0126^b^	0.17±0.0098^b^
	Jamapa	0.29±0.0097^a^	0.36±0.017^a^	0.28±0.01784^a^
g_c_ (mol m^-2^ s^-1^)	Calima	0.18±0.0064^b^	0.20±0.0107^b^	0.15±0.0083^b^
	Jamapa	0.26±0.0088 ^a^	0.31±0.01^a^	0.24±0.013^a^
WUE (μmol mmol^-1^)	Calima	1.50	3.46	5.13
	Jamapa	2.63	2.63	3.41

Conductance to CO_2_ (g_c_) was calculated using the formula g_c_=A/(Ca-Ci) Boyer and Kawamitsu (2011). n = 60. For each parameter, a different letter within a column indicates significant differences based on the Welch’s t-test at an alpha of 0.05 comparing both genotypes. The same letter indicates no significant differences.

Like g_sw_, stomatal conductance to CO_2_ (g_c_) increased in both genotypes as C_a_ increased from low to mid-level, but dropped significantly when C_a_ reached the highest level (**Table 2**). Unlike E rates, CO_2_ assimilation rates (A) increased to the highest level in both genotypes as CO_2_ levels increased (**Table 1**). Jamapa displayed statistically significant higher A rates than Calima at low and mid C_a_ levels, however, these differences disappeared at the highest C_a_ as C_i_ reached saturation in both genotypes, although Jamapa showed statistically higher C_i_ values than Calima at all C_a_ levels. The intercellular CO_2_ concentrations (C_i_) increased in both genotypes proportionally to the increases in C_a_ maintaining a Ci/Ca ratio of around 0.8. However, this ratio was lower in Calima than in Jamapa, particularly at the mid and high C_a_ levels. In summary, the response pattern of E, C_i_, A, g_sw_, and g_c_ to different levels of C_a_ was similar in both genotypes, but Jamapa displayed significantly higher values throughout. These differences could be explained largely by the fact that the total stomata aperture per unit of leaf area of Jamapa was 15% larger than that of Calima. However, Jamapa’s g_sw_ and g_c_ exceeded those of Calima’s by 44 to 78%. The disproportionality between total stomatal aperture per unit of leaf area and the estimated conductance strongly suggested other functional differences in addition to those of their epidermal anatomies.

**Table 2:** Leaf chlorophyll and protein characteristics between Calima and Jamapa.

Genotype	chlorophyll a (mg/m^2^)	chlorophyll b (mg/m^2^)	chlorophyll a/b ratio	Total chlorophyll (mg/m^2^)	Total protein (mg/m^2^)
Calima	108.0±3.76^b^	41.42±2.35^a^	2.82±0.19^a^	149.41±0.76^b^	399.90±25.84^b^
Jamapa	122.3± 3.95^a^	46.74±1.86^a^	2.65± 0.06^a^	169.02±1.13^a^	630.10±50.65^a^

Chlorophyll and total protein were measured in mature fully expanded leaves (n = 8). A different letter within a column indicates significant differences for each parameter based on Welch’s t-test at an alpha of 0.05 comparing both genotypes. The same letter indicates no significant differences.

An analysis of leaf-level water use efficiency (WUE) under the then atmospheric CO_2_ level (400 μmol mol^-1^) and possible future higher level (600 μmol mol^-1^) showed that Calima outperformed Jamapa by about 30%, an advantage that could increase to 50% under the high CO_2_ level (**Table 1**).

### Jamapa has higher chlorophyll and total protein content per unit area but carboxylation reactions in Calima are more efficient

We measured chlorophyll and protein content per unit of leaf area to investigate whether the differences in cell size between the genotypes could give rise to differences in the density of components of the photosynthetic apparatuses; these differences, if any, could also explain to some extent differences in photosynthetic capacities. Protein analysis indicated that there were statistically significant differences between Calima (399.9 mg/m^2^) and Jamapa (630.10 mg/m^2^) (**Table 2**). We found that both genotypes had similar chlorophyll a/b ratios: Calima (2.82) and Jamapa (2.65). However, the total chlorophyll content in Jamapa (169.02 mg/m^2^) was significantly higher than Calima’s (149.41 mg/m^2^) (**Table 3**).

**Table 3:** Carboxylation and electron transfer efficiencies of an Andean and a Mesoamerican common bean genotypes on total protein per unit area basis.

Genotype	A-Ci			
	V_cmax_ (μmol g^-1^ s^-1^)	J_max_ (μEq g^-1^ sec^-1^)	R_d_ (μmol g^-1^ s^-1^)	RuBP_sa-li_ (μmol mol^-1^)
Calima	167.8±1.44^a^	137.9±2.68^a^	4.72±0.19^a^	55.56±4.01^b^
Jamapa	125.0±0.26^b^	251.9±1.12^b^	3.31±0.03^b^	259.0±1.17^a^

The carboxylation efficiency on a total protein basis was estimated at moderate light (1000 µmol m^-2^ s^-1^ PPFD). Maximum carboxylation (V_cmax_), Maximum rate of photosynthetic electron transport (J_max_), rate of dark respiration (R_d_) was estimated by fitting the A-Ci curves from photosynthesis data collected from Calima and Jamapa, where CO_2_ was the substrate in the reaction adopting the Farquhar—von Caemmerer—Berry; FvCB model for C3 photosynthesis as implemented by Duursma (2015). Photosynthesis data was obtained under a standard light intensity of 1000 μmol m^-2^ s^-1^ PPFD, temperature of 25°C, and relative humidity of 60%. (n = 9). For each parameter, different letter indicates significant differences based on the Welch’s t-test at an alpha of 0.05 comparing both genotypes.

**Figure 6:**
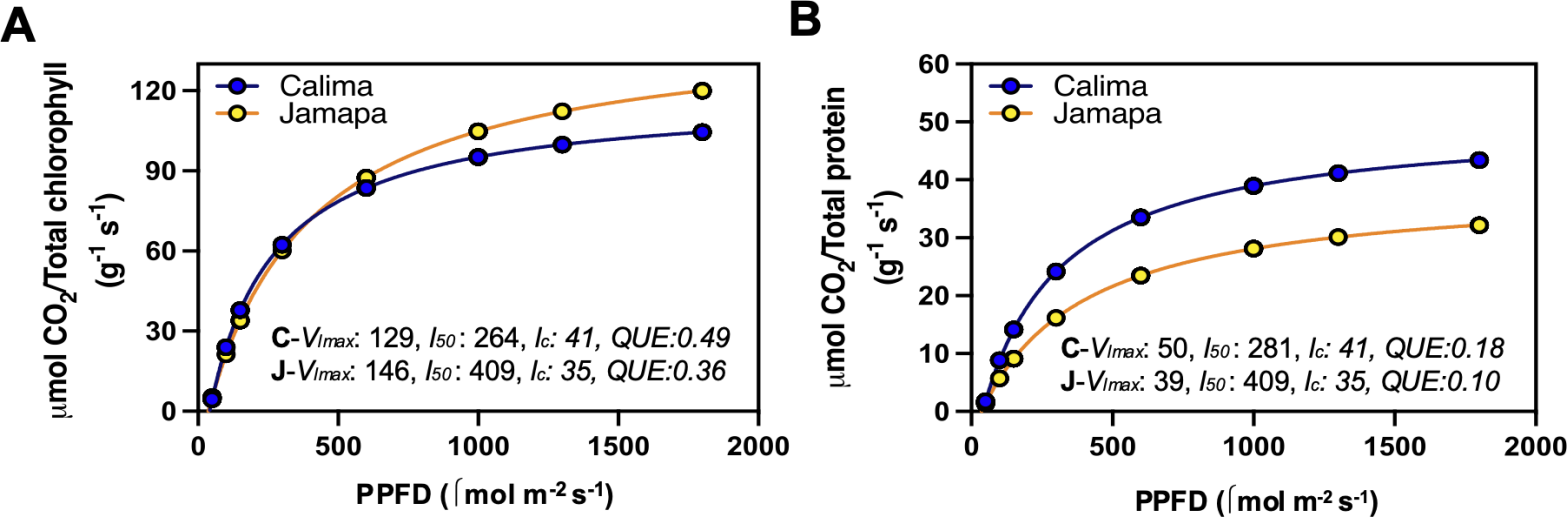
Photosynthetic light use efficiency after accounting for differences in chlorophyll and protein content between Andean and Mesoamerican common bean genotypes. LRCs from photosynthesis estimated per unit chlorophyll **(A)** and unit protein **(B)** from Calima and Jamapa. Fitting a modified hyperbolic function from light response curve (LRCs) data from Calima and Jamapa to estimate the light compensation points (l_c_; x-axis intercept), maximum photosynthetic rate at light-saturating conditions (V_lmax_; horizontal asymptote), the l_50_ or PPFDs needed to attain 0.5 V_lmax_ and an estimated quantum use efficiencies (QUE) by calculating the first derivative of the light function at l_c._ These estimates were from data collected between 8 am to 2.00 pm during the day at ambient CO_2_ 400 µmol mol^-1^. n = 36.

After estimating chlorophyll and protein content per unit of leaf area, we recalculated A_net_ based on either mg of protein or mg of chlorophyll instead of leaf area. Next, we fitted a modified hyperbolic function for the light response curves (LRCs) of Calima and Jamapa to compare their patterns of light dependance on net CO_2_ assimilation as a function of chlorophyll content (**Figure 6A**) and protein content (**Figure 6B**). After normalizing A_net_ for chlorophyll content (**Figure 6A**), Jamapa’s V_lmax_ (129) and l_50_ (264) were higher than Calima’s V_lmax_ (146) and l_50_ (409), but Calima displayed a higher l_c_ value (41) than Jamapa’s (35). In contrast, after normalizing A_net_ for protein content (**Figure 6B**), Calima’s V_lmax_ (50) was significantly higher than Jamapa’s V_lmax_ (39), but the estimated l_50s_ and l_cs_ remained almost unchanged for both genotypes (**Figure 6**). In general, the estimated QUE values were higher in both genotypes when calculated based on chlorophyll content than when calculated on protein basis. This is not surprising because light harvesting is primarily carried out by chlorophyll. Interestingly though, regardless of how A_net_ was expressed, Calima appeared to have a higher QUE value than Jamapa.

Considering this difference, we calculated the maximum rate of CO_2_ fixation based on protein content. Accordingly, Calima’s V_cmax_ rate (167.8 μmol g^-1^ sec^-1^) was 26% greater than Jamapa’s (125.0 μmol g^-1^ sec^-1^) (**Table 3**). However, when the results were expressed on a leaf area basis, Jamapa exceeded Calima by 12%. Regarding the electron transport efficiency J_max_, Jamapa (251.9 μEq g^-1^ sec^-1^) retained significantly higher values than Calima (137.9 μEq g^-1^ sec^-1^). The rate of dark respiration remained higher in Calima (4.72 μmol g^-1^ s^-1^) compared to Jamapa (3.31 μmol g^-1^ s^-1^). At the same time, the intercellular CO_2_ concentration at which these plants switched from carboxylation-limited to RuBP-limited was lower in Calima (55.56 μmol mol^-1^) and significantly higher in Jamapa (259 μmol mol^-1^) (**Table 3**). These results indicated that the carboxylation reactions in Calima are more efficient than those in Jamapa, while photosynthetic electron transport efficiency is higher in Jamapa.

## Discussion

Our analysis showed that the Mesoamerican bean (Jamapa) had higher photosynthesis at light-saturating conditions with a V_lmax_ that was 22% greater than that of the Andean bean (Calima) (**Figures 1**, **2**). Furthermore, this genotype exhibited a higher carboxylation efficiency with a V_cmax,_ which was 12% greater than that of Calima (**Figure 3**). However, when the V_cmax_ was expressed on the basis of total protein instead of leaf area, Jamapa’s advantage was nullified, and Calima had more efficient carboxylation reactions. These comparisons suggested that these genotypes have comparable photochemical capacities, but the structural differences that control CO_2_ diffusion, protein and chlorophyll content per unit of leaf area have a significant effect on their photosynthetic capacities.

The selection of a specific plant age was based on the observation that leaf anatomy and morphology normally changes during plant development (Wu and Poethig, 2006). In addition, several reports have documented increased phenotypic plasticity of leaves in older plants (Dorken and Barrett, 2004; Barton, 2008; Niinemets, 2016). Thus, we chose to use plants of similar age to ensure sample uniformity, and to avoid developmental factors increasing the number of variables for our study.

Several enzymes and proteins that function in photosynthesis are redox proteins whose activation changes with illumination and the circadian rhythm (Miyake et al., 2005; Thormählen et al., 2017; Cardona et al., 2018; Tan et al., 2021; Chen et al., 2022; Okegawa et al., 2022, 2023). As such, variable activation patterns in the redox proteins could explain the increasing trends in photosynthetic parameters from morning to the afternoon. However, in the afternoon, other factors could also affect the patterns, especially the stomatal closures to reduce the water loss, a situation likely to explain the drop in photosynthetic parameters (V_cmax_ and J_max_) of Calima compared to Jamapa. Other studies have shown that small stomata responded efficiently to fluctuating environments and could fine-tune gas exchange roles in limited conditions with less cost to the plant (Drake et al., 2013; Zhang et al., 2019). Smaller stomata at a higher density could contribute to higher rates of CO_2_ assimilation as it appeared to be the case for Jamapa. However, these features may become a liability under conditions of higher CO_2_ levels. In contrast to Jamapa, Calima showed a drastic increase in WUE at higher CO_2_ levels, which are expected as a result of climate change. These results suggest that we must examine in greater details the components of stomata conductance, such as stomata density and their responsiveness to external CO_2_ concentrations, when considering the development of cultivars for the future.

Our data showed that differences in stomatal and mesophyll cell sizes between the two genotypes had the most consequential effect on photosynthetic capacity. Calima had larger pavement cells than Jamapa which in effect lowered the stomatal densities on the abaxial and adaxial sides of the leaf (**Figure 4**). Evidence from other studies have indicated the importance of plant leaf anatomy on photosynthesis capacity. For instance, stomatal density controls the flow of CO_2_ into the intercellular spaces (Tomás et al., 2013; Harrison et al., 2020), as a result higher stomatal density promotes a better stomatal conductance (g_s_) compared to lower densities (Harrison et al., 2020). Furthermore, other studies have also shown that the stomatal density and stomata sizes are essential in fine-tuning the CO_2_ flow and managing the plant water balance (Drake et al., 2013; Tanaka et al., 2013). While smaller stomata exhibited faster responses to the environment, larger stomata have been observed to lag in the opening and closing (Drake et al., 2013). Our results indicated significant differences in the sizes of the stomata between the two genotypes. The larger guard cells and stoma opening of Calima were not able to counteract the low CO_2_ diffusion caused by a low stomatal density of this genotype (**Figure 4**, **Table 1**). In summary, total stomatal openings in Jamapa’s abaxial and adaxial leaf sides were 13% and 8% greater than in Calima’s, significantly contributing to enhanced differences in CO_2_ diffusion between the two plants.

As expected, the differences in cell sizes and stomatal apertures had a significant effect on stomatal conductance to water vapor and CO_2_. As a result of the higher stomatal conductance, Jamapa displayed greater rates of transpiration and CO_2_ assimilation than Calima. However, by the same token, Calima displayed greater WUE than Jamapa. This phenomenon should not be overlooked in light of climate change upon us where higher CO_2_ levels and temperatures are expected, conditions in which Calima is likely to have an advantage. Hence, Calima could be a source of unique genetic markers for breeding water use efficiency in common beans and other C3 legumes, especially under increasing challenges of inadequate water for agricultural production. However, substantial changes in stomatal conductance, especially through reduced stomatal density, could negatively impact assimilation, limiting the potential for higher benefits from this trait (Flexas, 2016; Ghannoum, 2016; Leakey et al., 2019; Israel et al., 2022). Our results are based on intrinsic water use efficiency (Flexas, 2016; Israel et al., 2022) and do not factor in the variation in leaf sizes of the genotypes and the total surface exposed for water loss.

Cell sizes are critical to mesophyll conductance in the diffusion of CO_2_ across several membranes into of chloroplast for CO_2_ fixation (Flexas et al., 2008; Tomás et al., 2013; Théroux-Rancourt and Gilbert, 2017; Elferjani et al., 2021; Momayyezi et al., 2022a). We also detected significant cell size differences in parenchyma and spongy mesophyll cells. Overall, Jamapa’s smaller cells resulted in larger cellular surface area per unit of leaf volume than Calima (**Figure 5**). In other studies, mesophyll cells’ surface area, density, and geometry affected the diffusion of CO_2_ into the chloroplast for photosynthesis (Flexas et al., 2008; Tomás et al., 2013; Peguero-Pina et al., 2017; Ren et al., 2019). Our results indicated that Jamapa mesophyll cells provided a larger area for CO_2_ diffusion into the cells than Calima cells. These results also suggested that the difference in chlorophyll content per unit of leaf area may be due to the differences in the size of mesophyll cells, provided the number and size of chloroplasts in the cells of each genotype are very similar.

There were differences in the size of pavement and guard cell between the abaxial and adaxial sides of the leaf in both genotypes. However, we noticed a lack of proportionality between genotypes. This result suggests that the developmental controls of the two sides could be independent to some extent. In addition, the differences in the number of pavement cells per stomata between genotypes also suggested another developmental polymorphism between the genotypes. Thicker leaves have previously been linked to an adaptation to lowlands in Juglans (Momayyezi et al., 2022b). Thus, longer palisade cells in Jamapa might be an adaptation to lower altitudes in the Mesoamerican region. Such thick leaves offer a double advantage of utilizing more light energy through increased number of chloroplasts and a higher mesophyll conductance (Momayyezi et al., 2022b). Therefore, leaf anatomical structure in Jamapa facilitates the efficient utilization of increasing light intensity, resulting in higher net photosynthesis. Therefore, while larger palisade and mesophyll cells surface area to volume ratio appear to be an adaptation to high light intensity, the opposite could be suitable for low light intensity.

Apart from the role of the anatomical differences in enhancing carboxylation rates in Jamapa, these differences also impacted their J_max_ throughout the day (**Figure 3**). Previous studies have shown that stomatal conductance is affected by the photosynthetic electron transport (Lawson et al., 2008), thus, impacting the PSI redox state (Li et al., 2021), changing the cyclic electron transport, influencing the NPQ system, and in general impacting the overall J_max_. Our results showed that Jamapa, which had higher stomatal density and smaller cell sizes, had higher stomatal conductance. In addition, these genotypes had significant differences in their chlorophyll content (**Table 2**). This agrees with previous studies that integrated chlorophyll content with the photosynthetic paraments and improved the empirical estimation of J_max_ from V_cmax_ (Song et al., 2021). Therefore, the better CO_2_ conductance in Jamapa could be explained by the anatomical and chlorophyll differences between the two genotypes and contributed to the high J_max_ stability in the afternoon.

A comparative analysis of Mesoamerican and Andean cultivars detected significant differences in organ size (Sexton et al., 1997). A cluster analysis of 427 bean genotypes from both gene pools documented that the main difference between the pools is yield potential, with the Mesoamerican lines excelling over the Andean lines (Amongi et al., 2023). The results presented here strongly suggest that the yield differences between the gene pools are most likely due to differences in photosynthetic capacity as influenced by their differences in anatomic characteristics. Furthermore, the availability of a genotyped recombinant inbred family produced between Jamapa and Calima (Bhakta et al., 2015), will facilitate the genetic analysis of cell size and testing of the hypothesis that cell size exerts significant control over photosynthetic performance.

## Data Availability

The original contributions presented in the study are included in the article/**Supplementary Material**. Further inquiries can be directed to the corresponding author.
