# Supplementary material for: Cell size differences affect photosynthetic capacity in a Mesoamerican and an Andean genotype of Phaseolus vulgaris L
Source: Front Plant Sci. 2024 Sep 11;15:1422814. doi: 10.3389/fpls.2024.1422814 (PMC11425597; doi:10.3389/fpls.2024.1422814)
Supplement: Supplementary file 1 [file DataSheet1.pdf]

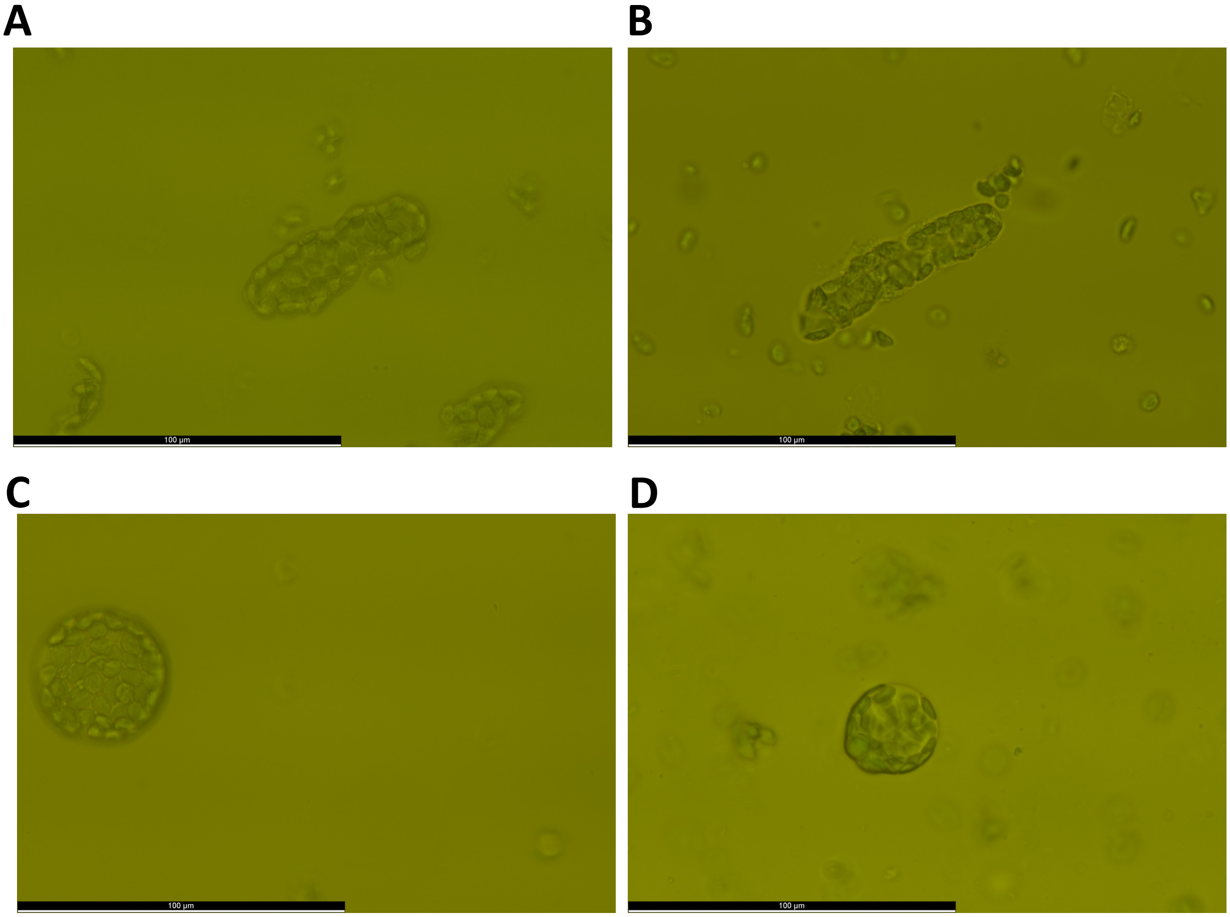

Supplementary Figure S1. Palisade and Mesophyll cell isolates from common bean leaves used in the study. Palisade cells from (A) Calima and (B) Jamapa. Mesophyll cells (C) Calima and (D) Jamapa. Isolation of Palisade and Mesophyll cells was done using a modified protoplast isolation protocol.
